# Serum Albumin, Globulin and Albumin–Globulin Ratios as Biomarkers of Clinical Outcomes in COVID-19 Pneumonia

**DOI:** 10.3390/jpm16060336

**Published:** 2026-06-22

**Authors:** Rauno Joks, Tamar Smith-Norowitz, Shawn Mathew, Mansi R. Kothari, Sairaman Nagarajan

**Affiliations:** 1 Department of Medicine, Kings County Hospital Center, SUNY Downstate Health Sciences University, 450 Clarkson Ave., Brooklyn, NY 11203, USA; 2 Department of Pediatrics, SUNY Downstate Health Sciences University, Brooklyn, NY 11203, USA; 3 Department of Pathology, SUNY Downstate Health Sciences University, Brooklyn, NY 11203, USA

**Keywords:** albumin, albumin/globulin ratio (AGR), COVID-19, personalized biomarker

## Abstract

**Objective**: Low serum albumin has been linked to morbidity and mortality in critically ill patients, including those with COVID-19. Whether serum globulin levels and albumin/globulin ratios (AGRs) can serve as biomarkers in COVID-19 is less well-characterized. This study assessed serum total protein, albumin, globulin levels, and AGR in relation to clinical outcomes in adults hospitalized with COVID-19 pneumonia. **Methods**: A retrospective EMR analysis was conducted among 569 hospitalized patients with COVID-19 pneumonia identified during the study period, of whom 60 met inclusion criteria of the required clinical immunologic and laboratory data and comprised the final analytic cohort. Variables included demographics and laboratory markers (total protein, albumin, globulin, immunoglobulins, CRP, and IL-6). The study evaluated: (1) Charlson Comorbidity Index (CCI), (2) Charlson 10-Year Estimated Survival (C10YES), (3) clinical severity using the NEWS-2 score, (4) length of hospital stay (LOS), and (5) mortality. Spearman correlations, chi-square tests, and regression analyses were conducted. **Results**: Albumin was independently associated with CCI, C10YES, and LOS in adjusted models (*p* = 0.01, *p* = 0.004, and *p* < 0.001, respectively), as was AGR (*p* = 0.012, *p* = 0.006, and *p* = 0.024, respectively). Decreasing total protein levels were independently associated with higher NEWS-2 scores, lower C10YES, and longer LOS (*p* = 0.009, *p* = 0.042, and *p* < 0.001, respectively). Increasing age was associated with longer LOS after adjustment for sex and the other plasma proteins. Globulin levels were not associated with clinical outcomes. **Conclusions**: Lower serum total protein was associated with higher COVID-19 severity, and lower albumin and AGR were associated with greater morbidity, lower predicted survival, and longer LOS. Increasing age was associated with longer LOS. In patients hospitalized with COVID-19 pneumonia, albumin and AGR may serve as potential biomarkers facilitating personalized risk stratification of hospital course and recovery.

## 1. Introduction

Since the onset of the Coronavirus disease (COVID-19) pandemic in 2020, there has been interest in understanding and assessing the underlying immunopathology in patients admitted with severe and critical disease caused by the SARS-CoV-2 virus [1]. The underlying pathophysiology and immune response after SARS-CoV-2 infection may affect the clinical course of COVID-19 infection and impact clinical outcomes [2]. While the pandemic has subsided, identifying affordable and readily available laboratory biomarkers from the COVID database can serve as indicators of inflammatory or immune responses may be of use in COVID and other viral outbreaks. These biomarkers should have immediate use in clinical settings for identifying the prognosis of hospitalized patients and in guiding clinical management approaches in the ongoing COVID epidemic.

Although no single biomarker can determine prognosis, plasma proteins such as albumin and globulin are indicators of hepatic synthetic function, and as such, reflect the impact of disease on the liver’s ability to maintain circulatory and immune homeostasis [3]. These proteins have been associated with numerous late-stage inflammatory diseases [4,5,6], and more recently with severe and critical COVID-19 [7]. Studies by Ulloque-Badaracco and colleagues have identified that serum albumin predicts COVID-19 severity and prognosis [8]. Albumin, a major protein in human plasma, plays a crucial role in maintaining oncotic pressure and regulating vascular permeability [3]. However, globulin levels (comprising serum immunoglobulins (Igs) called gamma globulins), provide humoral immunity to invading pathogens, including SARS-CoV-2 [9]. As an index measure, the ratio of albumins/globulins (AGR) can provide information about the balance between protein synthesis and degradation occurring in the body [10]. A meta-analysis found that low AGR was associated with increased severity and poor outcomes in COVID-19 pneumonia patients and may be a byproduct of the systemic inflammation and oxidative stress that occurs in response to the virus in critically ill patients [8]. Furthermore, the observation that low AGR levels can independently predict mortality in COVID-19 pneumonia patients [7] highlights their potential value in the identification of individuals with COVID-19 who are at a greater risk for unfavorable outcomes.

Previous studies in our laboratory demonstrated that serum Igs, specifically IgM, can serve as a potential biomarker of decreased morbidity and mortality, whereas IgG was associated with length of hospitalization in patients admitted with acute SARS-CoV-2 pneumonia [11]. To our knowledge, however, no prior study has concurrently evaluated total protein, albumin, globulin, and AGR in relation to multiple clinical outcomes within the same cohort of adults hospitalized with COVID-19 pneumonia, particularly with specific attention to globulin as a separate biomarker. The objective of this study was to assess whether total serum proteins, albumin, globulin, and the AGR could serve as personalized and actionable biomarkers associated with the aforementioned clinical outcomes in adults hospitalized with COVID-19 pneumonia.

## 2. Materials and Methods

### 2.1. Study Design and Data Source

We conducted a retrospective analysis of medical records at Kings County Hospital, a facility that is part of the New York City Health and Hospitals Corporation located in Brooklyn, New York. Study participants were residents of the surrounding catchment areas who were admitted by a clinician for inpatient management after presentation to the Kings County Emergency Department. Patients were admitted with COVID-19-related pneumonia (December 2020–April 2021), and documented using admission ICD-10 diagnosis codes J12.81, J12.82, and U07.1. These patients were identified from the EPIC database (electronic medical record, Epic Systems Corporation; Verona, WI, USA) and included in this study. Informed consent requirements were waived by the Institutional Review Boards due to it being a retrospective chart review. The study was conducted in accordance with the Declaration of Helsinki and approved by the Institutional Review Board of SUNY Downstate Medical Center (Protocol code 1843325, approved 14 January 2022) and Kings County Hospital Center (Protocol code 00003465, approved 21 June 2022). 

### 2.2. Inclusion Criteria

Patients ≥18 years of age who were hospitalized with documented SARS-CoV-2 infection and laboratory-confirmed positive real-time reverse transcriptase–polymerase chain reaction (RT-PCR) (nasopharyngeal (NP) swab) (Cobas SARS-CoV-2 RT-PCR test; Roche Molecular Systems, Inc., Pleasanton, CA, USA), as previously described [12], were eligible for inclusion. The final analytic cohort was further defined by availability of the required clinical biomarker data for the present analysis. No separate predefined exclusion criteria were applied beyond data availability for the variables of interest.

### 2.3. Data Collection

Demographics. Information on demographic variables including age and sex were collected. Since all patients in this sample/study population were from the same racial background (African American/Black), race/ethnicity was not used as a confounder in regression models.

Clinical factors. Other clinical variables collected included: vaccination status and information on concurrent comorbidities including allergy/asthma, cardiopulmonary disease, HIV status, and cancer, from which the Charlson Comorbidity Index and 10-year estimated survival (percent) [13] were computed (see below). Information on administration of remdesivir, tocilizumab, systemic corticosteroids and anticoagulation were included. All patients in this study received empiric antibiotic therapy; therefore, antibiotic use was not used as a confounder in the adjusted analyses.

Laboratory markers. Available total protein, serum albumin, serum globulin, and AGR were recorded (COBAS 8000, Roche Diagnostics, Indianapolis, IN, USA), as previously described [11].

Laboratory measurements were obtained as part of routine clinical care during hospitalization and were extracted from the electronic medical record. Detailed timing of CRP and IL-6 measurements relative to serum protein assessments, and precise temporal alignment of biomarker collection with clinical outcomes were not consistently available. Due to limited data, CRP and IL-6 were not considered for inclusion in the final analytic dataset.

### 2.4. Clinical Outcomes

We chose five clinical outcomes from the available clinical data: Charlson Comorbidity Index (CCI), Charlson 10-Year Estimated Survival (C10YES), length of stay (LOS), National Early Warning Score-2 (NEWS-2) score, and mortality. These were included to capture multiple clinically meaningful dimensions of patient status and hospital course, including comorbidity burden, estimated long-term survival, acute illness severity at presentation, duration of hospitalization, and in-hospital death. The method for calculating CCI and NEWS-2 scores has previously been described [14]. The C10YES was computed using the formula 0.983^(e^CCI×0.9^), with the caret sign “^” representing exponentiation. Because C10YES is derived from CCI, these measures were interpreted as related representations of the same underlying comorbidity construct rather than as statistically independent endpoints. LOS was calculated using the number of days between the dates of admission and discharge/ICU transfer/in-hospital mortality. Finally, a binary indicator of dying in-hospital (mortality) was created. Covariates were decided upon a priori due to potential clinical associations between primary independent and dependent variables and included sex for CCI and C10YES, and age and sex for the rest.

### 2.5. Statistical Methods

Descriptive and bivariate analyses on the demographic and clinical factors were conducted using Student’s t-test or non-parametric equivalents, stratified by level of outcome. Tests for normality were conducted using the Shapiro–Wilk test. Frequencies and percentages were computed for the categorical variables, also stratified by outcome status. Clinical outcomes were analyzed both continuously and categorically, except for mortality, which was only available categorized. When established, clinically validated thresholds available in the literature were used for categorization; for outcomes without established cutoffs, median-based dichotomization was used as an exploratory, distribution-driven approach to facilitate group comparisons and was not intended to represent clinically validated thresholds. The cutoffs for categorization were as follows: CCI: cutoff of 5 [15]; C10YES: cutoff of 80% [13]; NEWS-2: cutoff of 7 [16]; and LoS: cutoff was data-driven, with a median value of 10 days. The analytic cohort was restricted to patients with complete or near-complete data (<5% of sample missing) for the variables included in each analysis; no imputation procedures were performed.

Wherever possible, clinical variables were kept continuous; however, for ease of clinical interpretation, some descriptive analyses were conducted on categorized outcomes. Correlation analyses were performed among all laboratory variables, vaccination status, and our clinical outcomes using either Pearson or Spearman coefficients as appropriate for data distribution. Multivariable linear regression was used for continuous outcomes and logistic regression for mortality. Given the exploratory nature of this retrospective analysis and the limited sample size, findings from these models will be interpreted as associative rather than predictive. Advanced regression performance metrics and formal assessment of regression assumptions were not systematically performed. For all analyses, the level of statistical significance was pre-determined to be α = 0.05. To compute optimized clinical cutoffs and determine diagnostic performance (i.e., sensitivities and specificities) of AGR, receiver operating characteristic (ROC) curves with plot coordinates were generated for CCI, C10YES, NEWS-2, LoS, and mortality. All analyses were conducted using SPSS v26.0 (IBM SPSS Inc., Chicago, IL, USA). Area under the curve (AUC) values with 95% confidence intervals were calculated to quantify discriminative performance, and approximate optimal cutoffs were identified using ROC coordinate analysis.

## 3. Results

### 3.1. Patient Demographics

Hospital records were reviewed for adult patients who were hospitalized for acute COVID-19 pneumonia symptoms between December 2020 and April 2021 (N = 569). From those patients, N = 60 were selected, with available clinical immunologic evaluation data including total serum Ig levels, which had been drawn to assess humoral reserve. Their demographic data are listed in Table 1. The median age of the subjects (N = 60) was 61.7 ± 15.0 years; 58.3% were females and 41.7% were males. The mean total protein level was 7.02 ± 0.84 g/dL. Mean albumin, globulin, and AGR were 3.78 ± 0.61, 3.24 ± 0.49 g/dL and 1.19 ± 0.23 respectively. Before arriving at the emergency department, the subjects reported experiencing symptoms for a mean duration of 5.4 days ± 3.5. In addition, 91.7% of the subjects received systemic corticosteroid treatment, while 73.3% received remdesivir antiviral therapy. The average length of stay (LOS) was 13.15 ± 10.20 days, and 90% (N = 54) of all patients survived hospitalization and were eventually discharged (Table 1).

### 3.2. Correlation Between Serum Proteins and AGR with Clinical Outcomes

Table 2 shows that increasing age correlated with increasing lengths of stay (ρ = 0.356, *p* = 0.003). However, increasing total protein levels correlated with decreased LOS, as they were inversely related (ρ = −0.455, *p* < 0.003). Albumin correlated with both CCI and C10YES, albeit in opposite directions, i.e., increasing albumin correlated with decreased comorbidity (ρ = −0.302, *p* = 0.02) and increased estimated survival (ρ = 0.303, *p* = 0.02). Albumin was also strongly negatively correlated with LOS (ρ = −0.402, *p* = 0.002). Increasing globulin did not correlate with CCI, C10YES or LOS, but did weakly negatively correlate with NEWS-2 score (ρ = −0.261, *p* = 0.046). Similar to albumin, the AGR correlated with CCI (ρ = −0.278, *p* = 0.033) and C10YES (ρ = 0.281, *p* = 0.031) in opposite directions, but the strength of correlation was weaker. None of the laboratory markers showed any correlation with the likelihood of dying in-hospital in this sample. CRP and IL-6 did not correlate with any of our clinical outcomes.

### 3.3. Comparison of Serum Protein Levels and AGR Across Clinical Outcomes

As seen in Table 3, the mean levels of total protein (7.22 ± 0.51 vs. 6.35 ± 1.17, *p* = 0.034), albumin (3.92 ± 0.38 vs. 3.17 ± 0.88, *p* = 0.019), and AGR (1.21 ± 0.21 vs. 1.00 ± 0.27, *p* = 0.031) were all significantly higher in those with low CCI compared to those with high CCI. However, these differences did not attain statistical significance when measured across levels of low and high C10YES.

Mean total protein (7.25 ± 0.52 vs. 6.75 ± 0.97, *p* = 0.038) and albumin levels (3.94 ± 0.38 vs. 3.52± 0.76, *p* = 0.023) were also significantly higher in those with longer LOS. No significant difference was noted for globulin levels for any clinical outcome. There were no significant differences in the mean levels of any of the lab markers across levels of high/low NEWS-2 scores or mortality status (Table 3).

### 3.4. Regression Analysis of Demographics and Clinical Factors 

***CCI***. All adjusted regression models are shown in Table 4. Decreasing albumin (β = −0.339, *p* = 0.010) and decreasing AGR (β = −0.330, *p* = 0.012) were significantly associated with higher CCI. Decreasing total protein levels showed a non-significant trend toward association with higher CCI (β = −0.244, *p* = 0.074). Sex and globulin did not predict CCI.

***C10YES***. Increasing total protein levels (β = 0.273, *p* = 0.042), increasing albumin (β = 0.373, *p* = 0.0004) and increasing AGR (β = 0.330, *p* = 0.012) were associated with higher estimated survival as measured by C10YES. As above, sex and globulin did not impact estimated survival.

***NEWS-2 Score***. Decreasing total protein (β = −0.363, *p* = 0.009), but not AGR, was associated with high NEWS-2 scores, i.e., increased clinical severity on presentation (12), in models adjusted for age and sex. It is worth noting that decreasing albumin and globulin were marginally significant predictors (*p* = 0.058 and *p* = 0.053, respectively).

***LOS***. Higher age was significantly associated with length of hospital stay after adjusting for sex, and, independently, total protein levels, globulin levels, and AGR. Decreasing total protein levels were also associated with LOS after adjustment for age and sex (β = −0.493, *p* < 0.001), as were decreasing albumin (β = −0.530, *p* < 0.001) and decreasing AGR levels (β = −0.295, *p* = 0.024).

***Death***. In logistic regression models, dying in-hospital was not predicted by any of the protein levels or AGR; however, having higher total protein was marginally associated with lower odds of dying (OR = 0.404, 0.144–1.132. *p* = 0.085).

It is worth noting that the unadjusted correlations between key variables and outcomes are presented in Table 2, while Table 4 builds on this by evaluating their independent associations after adjustment for clinical covariates and determining whether the direction and significance of associations held after covariate adjustment.

Finally, as a post hoc correlation analysis, partial COVID-19 vaccination status (reported in 10% of patients) was not associated with serum protein levels or AGR, but showed weak yet significant correlations with higher CCI (ρ = 0.284, *p* = 0.028) and lower C10YES (ρ = −0.301, *p* = 0.019), without association with length of stay or in-hospital death.

**Figure 1:**
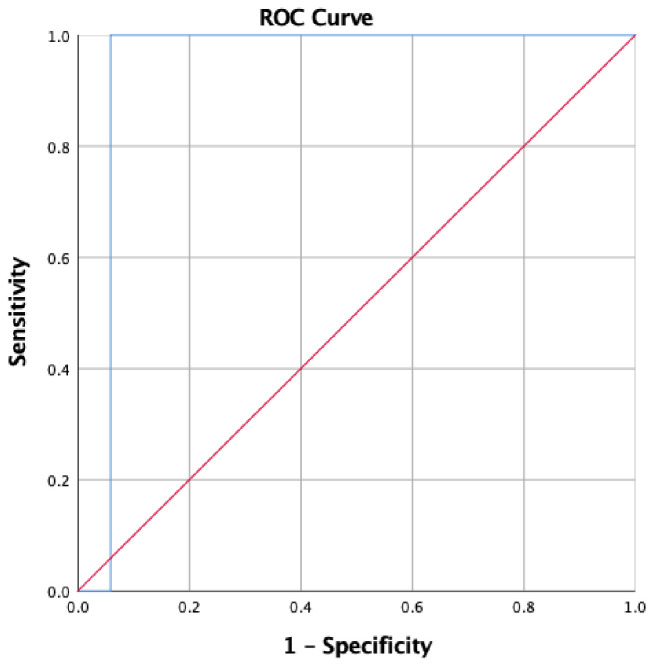
Receiver operating characteristic (ROC) curve for albumin–globulin ratio (AGR) predicting length of hospitalization. The curve demonstrates strong discriminative performance (AUC = 0.941), with cutoff values derived from coordinate analysis.

Post hoc receiver operating characteristic (ROC) analyses were performed to evaluate the discriminative performance of the albumin–globulin ratio (AGR) across clinical outcomes. We identified an approximate optimal AGR cutoff of 1.20, yielding 100% sensitivity and ~99% specificity, consistent with the performance of a near-perfect diagnostic test (Figure 1). At the same cutoff, predictive performance for other outcomes was as follows: CCI (69.2% sensitivity, 61% specificity), C10YES (46%, 46%), NEWS2 (62%, 58%), and mortality (100%, 60%). AGR demonstrated excellent discrimination for length of hospitalization (AUC = 0.941, 95% CI: 0.862–1.000, *p* < 0.001). For in-hospital mortality, AGR showed good discriminative ability (AUC = 0.829, 95% CI: 0.709–0.948, *p* = 0.009). In contrast, discriminative performance was modest for Charlson Comorbidity Index (AUC = 0.681, 95% CI: 0.535–0.828, *p* = 0.047) and poor for NEWS-2 score (AUC = 0.538, 95% CI: 0.359–0.718, *p* = 0.674). Charlson 10-Year Estimated Survival (C10YES) showed limited discrimination (AUC = 0.545, 95% CI: 0.394–0.696, *p* = 0.567). AUC values are summarized in Table 5, and ROC coordinate data for length of hospitalization are provided in Table 6. AUC and ROC tables for all outcomes are provided as supplementary information in Supplementary Materials.

## 4. Discussion

The results of this study indicate that higher total protein, albumin, and AGR levels were associated with lower comorbidity, improved predicted survival, and shorter hospital stays. Thus, among the proteins, albumin and the AGR (unlike globulin) appear to be potential indicators of immune status and clinical outcomes in COVID-19 pneumonia. Even though the CDC officially declared the COVID-19 pandemic over (11 May 2023), the need to study potential prognostic indicators of clinical outcomes is still germane to facilitate future pandemic and emergency preparedness. Recent studies in our laboratory identified IgM to be a biomarker of both decreased morbidity and mortality, with IgG associated with LOS in adult inpatients with COVID-19 pneumonia [11].

Albumin makes up more than half of the serum proteins in the blood and is important in the transport of bilirubin, hormones, metals, vitamins, and drugs [3]. Oxidized forms of albumin may have a role in immune signaling and may be related to the cytokine storm and overall systemic inflammation in COVID-19 [17]. Specifically, neutrophil reactive-oxygen-species oxidize serum albumin, which may upregulate pro-inflammatory signals interleukin-1 (IL-1), IL-6, and tumor necrosis factor (TNF) [18]. Despite their important role in COVID-19 pathophysiology, and the fact that albumin and globulin are easily measurable proteins, few studies [6,17] have investigated their role as a biomarker in patients with general COVID-19 outcomes or specific COVID-19 pneumonia.

Consistent with our findings, two studies from Wuhan, China demonstrated that serum albumin levels were significantly lower in non-survivors of COVID-19, and those with progressive and severe forms of COVID-19 [7,19]. Other studies have shown a relationship between low albumin and AGR and increased risk of severe disease [20,21,22,23,24], even in patients with concomitant COVID-19 and cancer [25], and those with acute respiratory distress syndrome (ARDS) [26]. In a prospective cohort study, a significant, negative, dose–response relationship of albumin in mild, severe, and critical COVID-19 patients was demonstrated [23]. Another study by Li and colleagues found that radiological severity, measured by CT scores, strongly negatively correlated with both albumin and AGR in a dose-dependent fashion [27]. In a study from Spain by de la Rica and colleagues, low albumin predicted both length of stay and mortality [28]. In another study from Italy, low albumin at admission identified patients at higher risk of severe respiratory failure, death, and longer LOS, but this study did not measure globulin or AGR [29].

In a meta-analysis of 11,356 patients corresponding to 31 cohort studies, patients with severe COVID-19 and those that died had much lower mean albumin levels than mild or survivor groups [8]. We found no published studies that assessed globulin levels separately from albumin and the AGR along with clinical outcomes, although one found an association of high globulin with all-cause mortality, albeit unrelated to SARS-CoV-2 [30]. Taken together, these studies suggest that both albumin levels and the AGR, but not globulin, may be associated with risk for poor outcomes.

Similar to our findings of age determining length of stay, increasing age is well-known to impact COVID-19 outcomes [31], and as such has been associated with clinical outcomes in several studies [7,20]. Age is an essential weighted component of the CCI and C10YES, with higher weights accorded to higher ages (ages 70–79 get 3 points and 80 and above get 4). Thus, age may be considered to indirectly influence these composite outcomes as well. It is worth noting that partial vaccination status was not significantly associated with the proteins, AGR or NEWS-2, LOS, or death in post hoc correlation analysis or chi-square tests. This was likely owing to the lack of vaccine availability, or access issues beyond rollout dates, compounded by vaccine hesitancy and skepticism in the urban minority population. However, partial vaccination correlated with both higher CCI and lower C10YES, suggesting that patients with more comorbidities may have been selected (either by themselves or by providers) to preferentially receive COVID-19 vaccines earlier, owing to their perceived higher risk.

Our findings agree with a previous meta-analysis evaluating the albumin–globulin relationship [7]. The observed associations between albumin/AGR and comorbidities, estimated survival, and LOS provide clinicians with actionable measurement tools to aid in decision-making regarding patient management, especially for those with systemic comorbidities. The correlation of CCI and C10YES with AGR is derived, in part, from globulin levels, which consist largely of total IgG [3]. In our previous study of inpatient adults with COVID-19 [11], increased total serum IgG was associated with increased age, LOS and worse initial clinical presentation requiring hospitalization. Prior studies in murine models have shown that IgG production is upregulated by interleukin (IL)-6 [32].

In humans, conditions associated with aging, including cardiovascular disease, are associated with increased blood levels of IL-6 [33]. IL-6, which increases with age [33], is linked to higher mortality and sarcopenia (age-related muscular atrophy), and can increase IgG, the predominant component of gamma globulin [34,35]. The age association with gamma globulin levels helps in understanding the utility of the albumin/globulin ratio as a biomarker. There is no correlation of total globulin alone with CCI or C10YES. However, lower albumin/globulin levels, which can result from a combination of decreased hepatic synthesis of albumin or albumin loss, along with increased gamma globulin (IgG) levels, does correlate with CCI and C10YES. Thus, the dual measurement of albumin, as liver synthetic output, with globulin (essentially IgG measurement), the latter as a marker of an aged immune response, does correlate with comorbidities and predicted survival.

Since albumin and AGR are routinely measured and easily determined, respectively, from most clinical laboratories and are very inexpensive, their use as potential biomarkers may provide a cost-effective way to identify disease outcomes and guide treatment decisions. This has particular relevance for hospitals, clinics and other healthcare institutions in resource-poor settings, or in lower- and middle-income countries (LMICs). An example of the beneficial utility of AGR in treatment algorithms would be consideration of use of strong immunosuppressive therapy, especially in older patients with poor nutritional status, whose lower AGRs correlate with lower survival. Use of less immunosuppressive treatment may align with greater survival in the older cancer patient. Future research should consider investigating the utility of albumin and AGR in combination with other clinical, radiological and laboratory parameters to develop more accurate predictive models of COVID-19 pneumonia outcomes, although work on this has already begun [24]. Finally, it is possible that albumin and AGR measurements may be evaluated as potential biomarkers in other infectious etiologies that cause reactive immune derangements and systemic inflammatory responses similar to that of the SARS-CoV-2 virus (i.e., pneumonia and acute respiratory distress syndromes).

Several limitations should be considered. First, this was a retrospective exploratory analysis of a relatively small analytic cohort, and the mortality outcome in particular was limited by low event counts. Second, laboratory timing relative to the clinical course was not consistently available, which limits temporal interpretation of associations involving serum proteins, CRP, and IL-6. Third, although models were adjusted for key demographic covariates, certain clinically relevant variables, including treatments, individual comorbidities, and inflammatory markers, were not included in all models because of incomplete or inconsistent availability in the analytic dataset, raising the possibility of residual confounding.

The ROC analysis further supports the clinical utility of AGR as a prognostic biomarker. AGR demonstrated strong discriminative ability for length of hospitalization and good performance for mortality, while performance for composite indices such as CCI, C10YES, and NEWS-2 was more modest. This variability likely reflects the multifactorial nature of composite clinical scores compared to directly measured outcomes. Importantly, the high sensitivity observed at selected AGR cutoff values suggests potential utility for screening or risk stratification in hospitalized patients. However, these findings should be interpreted with caution given the limited sample size, ties observed in ROC analyses, and the potential for overestimation of performance metrics.

There are several strengths to our study. This is the first study to our knowledge that links albumin, globulin and AGR measurements concurrently with quantified clinical outcomes. The choice of several clinical outcomes, including composite ones, adds biological plausibility to our findings.

Additionally, ROC analysis demonstrated that AGR has strong discriminative ability for length of hospitalization and moderate discriminative performance for mortality. However, these findings should be interpreted cautiously, as the relatively small sample size, presence of ties in ROC analyses, and potential overfitting may lead to overestimation of performance metrics.

## 5. Conclusions

These findings highlight the potential clinical utility of albumin and AGR as potential personalized and actionable biomarkers for COVID-19 morbidity and mortality. Further research is needed to confirm these findings and to explore the underlying mechanisms of the observed associations.

## Figures and Tables

**Table 1:** Patient demographics and clinical factors.

Patient Characteristics (N = 60)	N (%)
Age (years) *	61.73 ± 14.97
Gender	
Male	25 (41.7%)
Female	35 (58.3%)
Inhaled corticosteroid (ICS) use	
No	5 (8.3%)
Yes	55 (91.7%)
Tocilizumab use	
No	55 (91.7%)
Yes	5 (8.3%)
Systemic Anticoagulants	
No	9 (15%)
Yes	51 (85%)
Remdesevir	
No	16 (26.7%)
Yes	44 (73.3%)
Total serum protein (g/dL) *	7.02 ± 0.84
Albumin (g/dL) *	3.78 ± 0.61
Globulin (g/dL) *	3.24 ± 0.49
Albumin/Globulin Ratio *	1.19 ± 0.23
Duration of symptoms prior to admission (days) *	5.4 ± 3.48
NEWS-2 Score * (0–23)	5.63 ± 2.86
Charlson Comorbidity Index (CCI) * (0–33)	3.63 ± 2.66
Charlson 10-Year Estimated Survival Score (C10YES) *	59.27 ± 37.31
Length of stay (LoS) (days) *	13.15 ± 10.20
Mortality	
Survived/discharged alive	54 (90%)
Died in-hospital	6 (10%)

* Data reported as mean ± standard deviation (SD). In adults: normal range for total protein, albumin, globulin, and AGR. The average duration of hospital stay prior to testing for serum Ig was 4.66 ± 4.46 days. Note that the Charlson Comorbidity Index (CCI) is a validated scoring system that quantifies a patient’s overall comorbidity burden based on the number and severity of chronic diseases. Charlson Estimated 10-Year Survival (C10YES) is a percentage derived from the Charlson Comorbidity Index (CCI) that estimates a patient’s likelihood of surviving the next 10 years based on their comorbid conditions; accordingly, these two outcomes reflect related dimensions of the same underlying construct.

**Table 2:** Correlation coefficients for serum proteins, AGR, and clinical outcomes.

Serum Levels	CCI		C10YES		NEWS-2		LOS		Mortality	
Correlation	ρ	*p*-Value	ρ	*p*-Value	ρ	*p*-Value	ρ	*p*-Value	ρ	*p*-Value
Age	-	-	-	-	0.166	0.174	0.356	0.003	0.108	0.380
Total Protein	−0.190	0.148	0.190	0.150	−0.218	0.097	−0.455	<0.001	−0.109	0.417
Albumin	−0.302	0.020	0.303	0.020	−0.137	0.302	−0.402	0.002	−0.124	0.353
Globulin	0.026	0.844	−0.023	0.863	−0.261	0.046	−0.218	0.100	−0.049	0.714
AGR	−0.278	0.033	0.281	0.031	0.016	0.904	−0.170	0.203	−0.130	0.330
CRP	−0.157	0.270	0.152	0.287	0.005	0.972	−0.142	0.335	−0.232	0.102
IL-6	-	-	-	-	0.866	0.333	−0.500	0.667	-	-

Data represents Spearman correlation coefficients (ρ) reported with respective *p*-values to the right. Note that age was a weighted component of CCI and C10YES; therefore, correlations cannot be computed for these two associations. The “-“ in the IL-6 column denotes insufficient data for computing correlation coefficient. Abbreviations: CCI—Charlson Comorbidity Index. C10YES—Charlson 10-Year Estimated Survival, NEWS-2 –National Early Warning Score -2 Score, LOS—Length of stay, AGR—albumin–globulin ratio.

**Table 3:** Mean serum protein levels and albumin/globulin ratios across clinical outcomes.

Clinical Outcome	Total Serum Protein (g/dL)	*p*-Value	Albumin (g/dL)	*p*-Value	Globulin (g/dL)	*p*-Value	Albumin/Globulin Ratio	*p*-Value
Charlson Comorbidity Index (CCI)								
Low	7.22 ± 0.51	0.034	3.92 ± 0.38	0.019	3.30 ± 0.45	0.732	1.21 ± 0.21	0.031
High	6.35 ± 1.17		3.17 ± 0.88		3.17 ± 0.51		1.00 ± 0.27	
Charlson 10-Year Estimated Survival (C10YES)								
Low	6.94 ± 0.93	0.237	3.65 ± 0.63	0.069	3.29 ± 0.50	0.730	1.12 ± 0.23	0.096
High	7.17 ± 0.45		3.93 ± 0.55		3.24 ± 0.41		1.24 ± 0.23	
NEWS-2 Score								
Low	7.14 ± 0.54	0.904	3.86 ± 0.43	0.250	3.27 ± 0.35	0.133	1.20 ± 0.20	0.207
High	6.66 ± 1.36		3.34 ± 0.99		3.26 ± 0.79		1.05 ± 0.33	
Length of hospitalization (LOS)								
High	7.25 ± 0.52	0.038	3.94 ± 0.38	0.023	3.30 ± 0.39	0.584	1.21 ± 0.21	0.613
Low	6.75 ± 0.97		3.52 ± 0.76		3.23 ± 0.55		1.11 ± 0.26	
In-hospital mortality								
Discharged alive	7.12 ± 0.66	0.363	3.49 ± 0.52	0.351	3.27 ± 0.47	0.447	1.20 ± 0.22	0.338
Died	6.45 ± 1.62		3.38 ± 1.09		3.07 ± 0.59		1.08 ± 0.27	

Data reported as mean ± standard deviation (SD) with respective *p*-values to the right. Statistical tests for mean comparisons were independent sample t-tests with unequal variance. In adults: normal range for total protein, albumin, globulin, and AGR. Low Charlson Comorbidity Index (CCI) defined as ≤ 5, high CCI as > 5. Low Charlson 10-Year Estimated Survival (C10YES) defined as ≤80%, high C10YES as > 80%. Low NEWS-2 score defined as ≤7, high NEWS-2 as > 7. Low Length of Stay (LOS) defined as ≤10 days, high LOS as > 10 days.

**Table 4:** Regression analysis of demographics and clinical factors stratified by all outcomes.

Outcome/Parameter	β	Std. Error (S.E.)	*p*-Value
**Charlson Comorbidity Index**			
I. Total Protein	−0.244	0.448	0.074
Sex	0.085	0.735	0.529
II. Albumin	−0.339	0.568	0.010
Sex	0.101	0.800	0.427
III. Globulin	0.037	0.802	0.788
Sex	0.155	0.759	0.267
IV. Albumin:Globulin Ratio	−0.330	1.493	0.012
Sex	0.167	0.693	0.190
**Charlson 10-Year Estimated Survival**			
I. Total Protein	0.273	6.171	0.042
Sex	−0.139	10.107	0.292
II. Albumin	0.373	7.756	0.004
Sex	−0.159	9.496	0.203
III. Globulin	−0.033	11.128	0.809
Sex	−0.216	1.579	0.120
IV. Albumin:Globulin Ratio	0.355	20.478	0.006
Sex	−0.231	9.505	0.066
**NEWS-2 Score**			
I. Total Protein	−0.363	0.461	0.009
Age	0.041	0.026	0.760
Sex	−0.001	0.743	0.991
II. Albumin	−0.268	0.636	0.058
Age	0.042	0.028	0.766
Sex	0.053	0.752	0.691
III. Globulin	−0.266	0.798	0.053
Age	0.130	0.026	0.333
Sex	−0.002	0.775	0.990
IV. Albumin:Globulin Ratio	−0.022	1.692	0.876
Age	0.124	0.028	0.389
Sex	0.071	0.783	0.613
Length of Stay			
I. Total Protein	−0.493	1.508	<0.001
Age	0.242	0.085	0.045
Sex	−0.120	2.452	0.311
II. Albumin	−0.530	1.963	<0.001
Age	0.189	0.085	0.115
Sex	−0.054	2.342	0.631
III. Globulin	−0.157	2.874	0.238
Age	0.363	0.094	0.007
Sex	−1.296	2.820	0.648
IV. Albumin:Globulin Ratio	−0.295	5.670	0.024
Age	0.283	0.094	0.034
Sex	0.017	2.649	0.895
**Mortality**	**OR**	**95% Confidence Interval**	***p*-value**
I. Total Protein	0.404	0.144–1.132	0.085
Age	1.017	0.947–1.093	0.642
Sex	0.405	0.059–2.763	0.356
II. Albumin	0.372	0.107–1.295	0.120
Age	1.013	0.943–1.090	0.707
Sex	0.501	0.078–3.234	0.468
III. Globulin	0.252	0.030–2.097	0.202
Age	1.031	0.961–1.105	0.394
Sex	0.432	0.068–2.738	0.373
IV. Albumin:Globulin Ratio	0.182	0.005–6.876	0.358
Age	1.022	0.951–1.097	0.558
Sex	0.613	0.101–3.717	0.595

Multivariate linear regression analysis of demographic parameters and all Ig levels with each clinical outcome.

**Table 5:** Area under the curve values for albumin–globulin ratio (AGR) across clinical outcomes.

Categorized Outcome	AUC	95% CI	*p*-Value	Interpretation
Length of stay (LOS)	0.941	0.862–1.000	<0.001	Excellent
In-hospital mortality	0.829	0.709–0.948	0.009	Good
NEWS-2	0.538	0.359–0.718	0.674	Poor
Charlson Comorbidity Index	0.681	0.535–0.828	0.047	Modest
Charlson 10-Year Estimated Survival	0.545	0.394–0.696	0.567	Poor

**Table 6:** Diagnostic performance of albumin-to-globulin ratio (AGR) from receiver operating characteristic curve analysis (coordinates for LOS).

Coordinates of the Receiver Operating Curve for Albumin–Globulin Ratio		
Positive if Greater Than or Equal To ^a^	Sensitivity	1 − Specificity
1.01	1.000	0.706
1.03	1.000	0.647
1.03	1.000	0.618
1.04	1.000	0.588
1.07	1.000	0.559
1.09	1.000	0.529
1.10	1.000	0.500
1.11	1.000	0.471
1.12	1.000	0.412
1.12	1.000	0.382
1.13	1.000	0.353
1.14	1.000	0.294
1.16	1.000	0.265
1.17	1.000	0.235
1.17	1.000	0.206
1.18	1.000	0.176
1.18	1.000	0.147
1.19	1.000	0.118
1.20	1.000	0.088
1.20	1.000	0.059
1.22	0.960	0.059
1.23	0.880	0.059
1.25	0.840	0.059
1.27	0.800	0.059
1.28	0.720	0.059
1.30	0.680	0.059
1.31	0.600	0.059
1.33	0.560	0.059
1.34	0.520	0.059
1.35	0.480	0.059
1.36	0.440	0.059
1.38	0.400	0.059
1.40	0.320	0.059
1.40	0.280	0.059
1.44	0.240	0.059
1.48	0.200	0.059
1.48	0.160	0.059
1.48	0.120	0.059
1.51	0.080	0.059
1.55	0.000	0.059
1.63	0.000	0.029
2.70	0.000	0.000

**Note**: ^a^ The smallest cutoff (coordinate) value is the minimum observed test value minus 1, and the largest cutoff value is the maximum observed test value plus 1. All the other cutoff values are the averages of two consecutive ordered observed test values. Coordinates of the ROC curve are presented in the first column, with corresponding sensitivity and specificity values for AGR for the clinical outcome length of hospitalization. Coordinate values are rounded to two decimal places.

## Data Availability

The data presented in this study are available on request from the corresponding author. The data are not publicly available due to privacy restrictions.

## Supplementary Materials

The following supporting information can be downloaded at https://www.mdpi.com/article/10.3390/jpm16060336/s1, File S1: ROC AUC Analyses ALL outcomes.
