# Supplementary material for: Serum Albumin, Globulin and Albumin–Globulin Ratios as Biomarkers of Clinical Outcomes in COVID-19 Pneumonia
Source: J Pers Med. 2026 Jun 22;16(6):336. doi: 10.3390/jpm16060336 (PMC13302725; doi:10.3390/jpm16060336)
Supplement: Supplementary file 1 [file jpm-16-00336-s001.zip › jpm-4255120-supplementary.pdf]

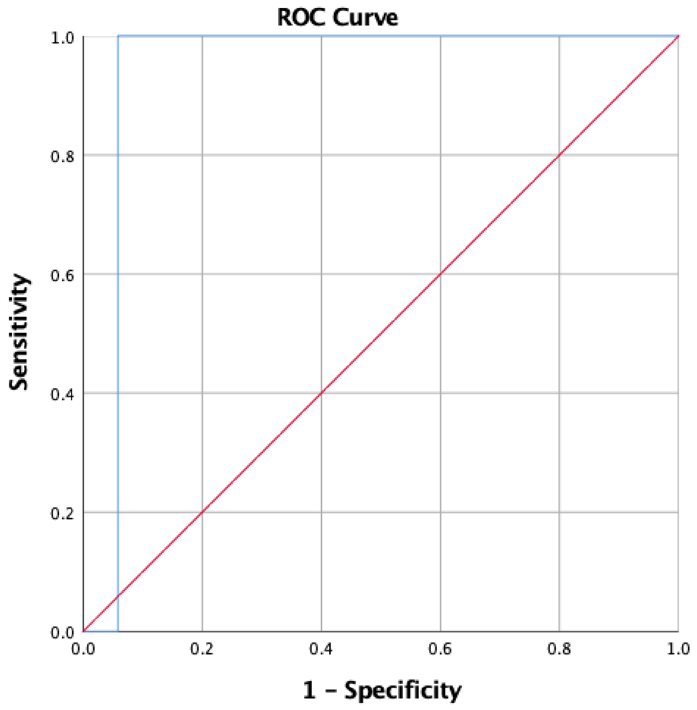

LOS

Area Under the Curve

Test Result Variable(s): Alb:glob

Area	Std. Error[a]	Asymptotic Sig.[b]	Asymptotic 95% Confidence Interval	
			Lower Bound	Upper Bound
.941	.040	.000	.862	1.000

a. Under the nonparametric assumption

b. Null hypothesis: true area = 0.5

Coordinates of the Curve

Test Result Variable(s): Alb:glob

Positive if Greater Than or Equal To[a]	Sensitivity	1 - Specificity

-.435897435897436	1.000	1.000
.582051282051282	1.000	.971
.714285714285714	1.000	.912
.833640552995392	1.000	.882
.868072787427626	1.000	.853
.920940170940171	1.000	.824
.958333333333333	1.000	.794
.986111111111111	1.000	.765
1.013513513513514	1.000	.706
1.027799227799228	1.000	.647
1.029437229437229	1.000	.618
1.044563279857397	1.000	.588
1.072268907563025	1.000	.559
1.094581280788177	1.000	.529
1.104355716878403	1.000	.500
1.111455108359133	1.000	.471
1.119429590017825	1.000	.412
1.123106060606061	1.000	.382
1.133928571428572	1.000	.353

1.144957983193277	1.000	.294
1.156862745098039	1.000	.265
1.169540229885058	1.000	.235
1.174442190669371	1.000	.206
1.179144385026738	1.000	.176
1.184659090909091	1.000	.147
1.190524193548387	1.000	.118
1.196774193548387	1.000	.088
1.204166666666667	1.000	.059
1.217069892473118	.960	.059
1.234115347018573	.880	.059
1.254545454545454	.840	.059
1.269696969696970	.800	.059
1.283422459893048	.720	.059
1.298573975044564	.680	.059
1.312805474095796	.600	.059
1.327956989247312	.560	.059
1.337719298245614	.520	.059
1.348471986417657	.480	.059

1.36075268 8172043	.440	.059
1.37976190 4761905	.400	.059
1.39642857 1428571	.320	.059
1.40370370 3703704	.280	.059
1.44179894 1798942	.240	.059
1.47883597 8835979	.200	.059
1.48212005 1085568	.160	.059
1.48331479 4215795	.120	.059
1.50979262 6728110	.080	.059
1.55357142 8571429	.000	.059
1.63354037 2670808	.000	.029
2.69565217 3913043	.000	.000

a. The smallest cutoff value is the minimum observed test value minus 1, and the largest cutoff value is the maximum observed test value plus 1. All the other cutoff values are the averages of two consecutive ordered observed test values.

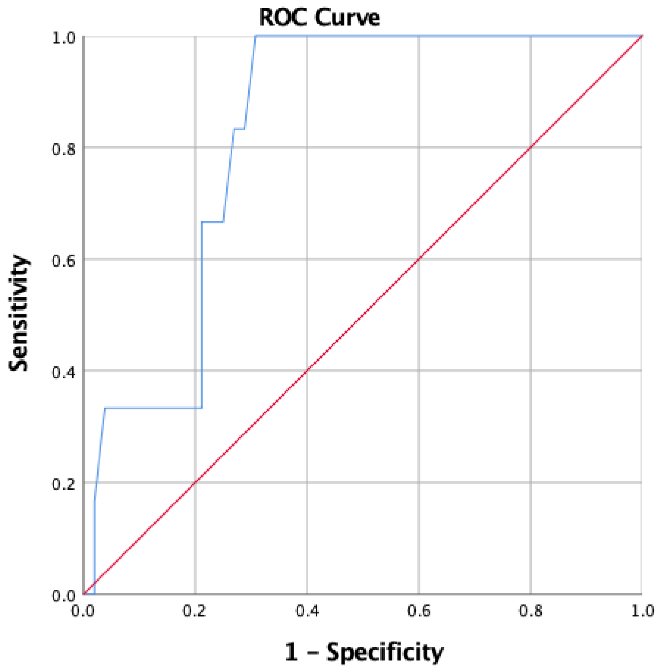

Diagonal segments are produced by ties.

DEATH

Area Under the Curve

Test Result Variable(s): Alb:glob

Area	Std. Error[a]	Asymptotic Sig.[b]	Asymptotic 95% Confidence Interval Lower Bound	Upper Bound
.829	.061	.009	.709	.948

The test result variable(s): Alb:glob has at least one tie between the positive actual state group and the negative actual state group. Statistics may be biased.

a. Under the nonparametric assumption

b. Null hypothesis: true area = 0.5

Coordinates of the Curve

Test Result Variable(s): Alb:glob

Positive if Greater Than or Equal To[a]	Sensitivity	1 - Specificity
-.435897435897436	1.000	1.000
.582051282051282	1.000	.981
.714285714285714	1.000	.942
.833640552995392	1.000	.923
.868072787427626	1.000	.904
.920940170940171	1.000	.885
.958333333333333	1.000	.865
.986111111111111	1.000	.846
1.013513513513514	1.000	.808
1.027799227799228	1.000	.769
1.029437229437229	1.000	.750
1.044563279857397	1.000	.731
1.072268907563025	1.000	.712
1.094581280788177	1.000	.692
1.104355716878403	1.000	.673

1.11145510 8359133	1.000	.654
1.11942959 0017825	1.000	.635
1.12310606 0606061	1.000	.615
1.13392857 1428572	1.000	.596
1.14495798 3193277	1.000	.558
1.15686274 5098039	1.000	.538
1.16954022 9885058	1.000	.519
1.17444219 0669371	1.000	.500
1.17914438 5026738	1.000	.481
1.18465909 0909091	1.000	.462
1.19052419 3548387	1.000	.442
1.19677419 3548387	1.000	.423
1.20416666 6666667	1.000	.404
1.21706989 2473118	1.000	.385
1.23411534 7018573	1.000	.346
1.25454545 4545454	1.000	.327
1.26969696 9696970	1.000	.308
1.28342245 9893048	.833	.288
1.29857397 5044564	.833	.269

1.31280547 4095796	.667	.250
1.32795698 9247312	.667	.231
1.33771929 8245614	.667	.212
1.34847198 6417657	.500	.212
1.36075268 8172043	.333	.212
1.37976190 4761905	.333	.192
1.39642857 1428571	.333	.154
1.40370370 3703704	.333	.135
1.44179894 1798942	.333	.115
1.47883597 8835979	.333	.096
1.48212005 1085568	.333	.077
1.48331479 4215795	.333	.058
1.50979262 6728110	.333	.038
1.55357142 8571429	.167	.019
1.63354037 2670808	.000	.019
2.69565217 3913043	.000	.000

The test result variable(s): Alb:glob has at least one tie between the positive actual state group and the negative actual state group.

a. The smallest cutoff value is the minimum observed test value minus 1, and the largest cutoff value is the maximum observed test value plus 1. All the other cutoff values are the averages of two consecutive ordered observed test values.

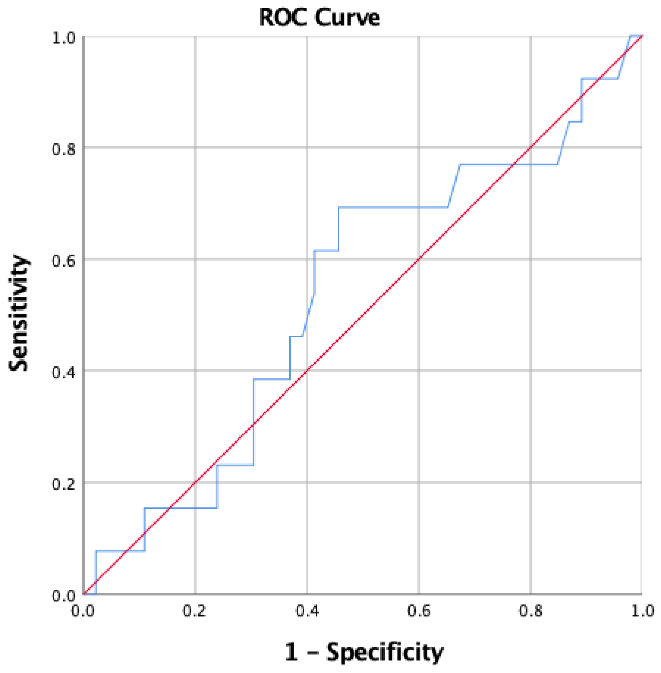

Diagonal segments are produced by ties.

NEWS2CAT

Area Under the Curve

Test Result Variable(s): Alb:glob

Area	Std. Error[a]	Asymptotic Sig.[b]	Asymptotic 95% Confidence Interval Lower Bound	Asymptotic 95% Confidence Interval Upper Bound
.538	.092	.674	.359	.718

The test result variable(s): Alb:glob has at least one tie between the positive actual state group and the negative actual state group. Statistics may be biased.

a. Under the nonparametric assumption

b. Null hypothesis: true area = 0.5

Coordinates of the Curve

Test Result Variable(s): Alb:glob

Positive if Greater Than or Equal To[a]	Sensitivity	1 - Specificity
-.435897435897436	1.000	1.000
.582051282051282	1.000	.978
.714285714285714	.923	.957
.833640552995392	.923	.935
.868072787427626	.923	.913
.920940170940171	.923	.891
.958333333333333	.846	.891
.986111111111111	.846	.870
1.013513513513514	.769	.848
1.027799227799228	.769	.804
1.029437229437229	.769	.783
1.044563279857397	.769	.761
1.072268907563025	.769	.739
1.094581280788177	.769	.717
1.104355716878403	.769	.696
1.111455108359133	.769	.674
1.119429590017825	.692	.652

1.123106060606061	.692	.630
1.133928571428572	.692	.609
1.144957983193277	.692	.565
1.156862745098039	.692	.543
1.169540229885058	.692	.522
1.174442190669371	.692	.500
1.179144385026738	.692	.478
1.184659090909091	.692	.457
1.190524193548387	.615	.457
1.196774193548387	.615	.435
1.204166666666667	.615	.413
1.217069892473118	.538	.413
1.234115347018573	.462	.391
1.254545454545454	.462	.370
1.269696969696970	.385	.370
1.283422459893048	.385	.326
1.298573975044564	.385	.304
1.312805474095796	.231	.304
1.327956989247312	.231	.283

1.33771929 8245614	.231	.261
1.34847198 6417657	.231	.239
1.36075268 8172043	.154	.239
1.37976190 4761905	.154	.217
1.39642857 1428571	.154	.174
1.40370370 3703704	.154	.152
1.44179894 1798942	.154	.130
1.47883597 8835979	.154	.109
1.48212005 1085568	.077	.109
1.48331479 4215795	.077	.087
1.50979262 6728110	.077	.065
1.55357142 8571429	.077	.022
1.63354037 2670808	.000	.022
2.69565217 3913043	.000	.000

The test result variable(s): Alb:glob has at least one tie between the positive actual state group and the negative actual state group.

a. The smallest cutoff value is the minimum observed test value minus 1, and the largest cutoff value is the maximum observed test value plus 1. All the other cutoff values are the averages of two consecutive ordered observed test values.

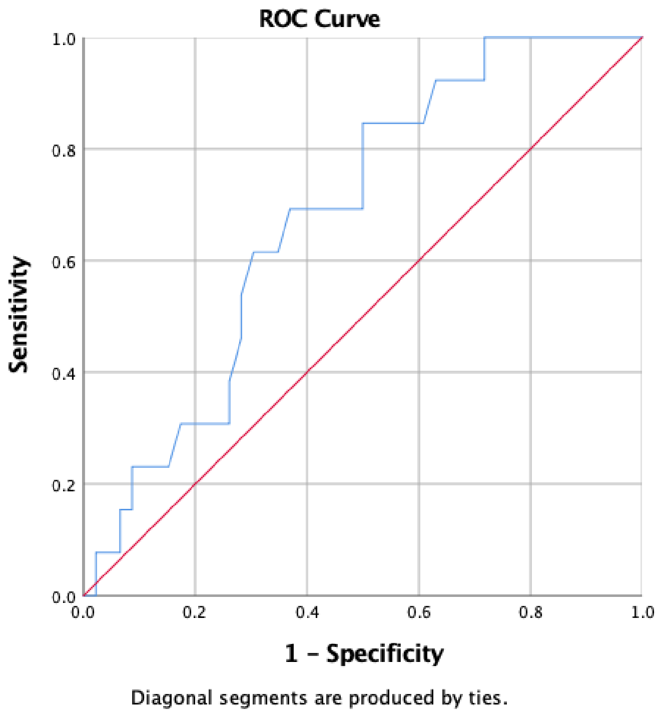

CCICAT

Area Under the Curve

Test Result Variable(s): Alb:glob

Area	Std. Error[a]	Asymptotic Sig.[b]	Asymptotic 95% Confidence Interval Lower Bound	Asymptotic 95% Confidence Interval Upper Bound
.681	.075	.047	.535	.828

The test result variable(s): Alb:glob has at least one tie between the positive actual state group and the negative actual state group. Statistics may be biased.

a. Under the nonparametric assumption

b. Null hypothesis: true area = 0.5

Coordinates of the Curve

Test Result Variable(s): Alb:glob

Positive if Greater Than or Equal To[a]	Sensitivity	1 - Specificity
-.435897435897436	1.000	1.000
.582051282051282	1.000	.978
.714285714285714	1.000	.935
.833640552995392	1.000	.913
.868072787427626	1.000	.891
.920940170940171	1.000	.870
.958333333333333	1.000	.848
.986111111111111	1.000	.826
1.013513513513514	1.000	.783
1.027799227799228	1.000	.739
1.029437229437229	1.000	.717
1.044563279857397	.923	.717
1.072268907563025	.923	.696
1.094581280788177	.923	.674
1.104355716878403	.923	.652
1.111455108359133	.923	.630
1.119429590017825	.846	.609

1.123106060606061	.846	.587
1.133928571428572	.846	.565
1.144957983193277	.846	.522
1.156862745098039	.846	.500
1.169540229885058	.769	.500
1.174442190669371	.692	.500
1.179144385026738	.692	.478
1.184659090909091	.692	.457
1.190524193548387	.692	.435
1.196774193548387	.692	.413
1.204166666666667	.692	.391
1.217069892473118	.692	.370
1.234115347018573	.615	.348
1.254545454545454	.615	.326
1.269696969696970	.615	.304
1.283422459893048	.538	.283
1.298573975044564	.462	.283
1.312805474095796	.385	.261
1.327956989247312	.308	.261

1.33771929 8245614	.308	.239
1.34847198 6417657	.308	.217
1.36075268 8172043	.308	.196
1.37976190 4761905	.308	.174
1.39642857 1428571	.231	.152
1.40370370 3703704	.231	.130
1.44179894 1798942	.231	.109
1.47883597 8835979	.231	.087
1.48212005 1085568	.154	.087
1.48331479 4215795	.154	.065
1.50979262 6728110	.077	.065
1.55357142 8571429	.077	.022
1.63354037 2670808	.000	.022
2.69565217 3913043	.000	.000

The test result variable(s): Alb:glob has at least one tie between the positive actual state group and the negative actual state group.

a. The smallest cutoff value is the minimum observed test value minus 1, and the largest cutoff value is the maximum observed test value plus 1. All the other cutoff values are the averages of two consecutive ordered observed test values.

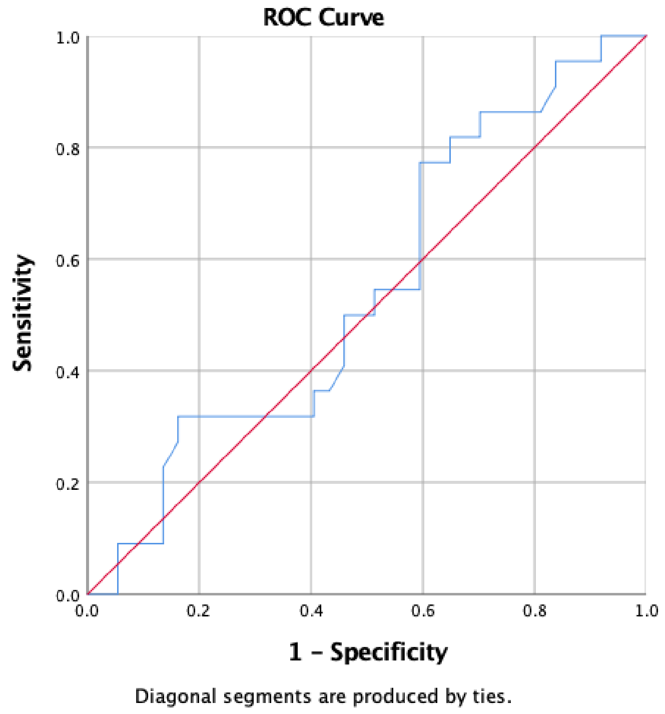

Area Under the Curve

Test Result Variable(s): Alb:glob

Area	Std. Error[a]	Asymptotic Sig.[b]	Asymptotic 95% Confidence Interval Lower Bound	Upper Bound
.545	.077	.567	.394	.696

The test result variable(s): Alb:glob has at least one tie between the positive actual state group and the negative actual state group. Statistics may be biased.

a. Under the nonparametric assumption

b. Null hypothesis: true area = 0.5

Coordinates of the Curve

Test Result Variable(s): Alb:glob

Positive if Greater Than or Equal To[a]	Sensitivity	1 - Specificity
-.435897435897436	1.000	1.000
.582051282051282	1.000	.973
.714285714285714	1.000	.919
.833640552995392	.955	.919
.868072787427626	.955	.892
.920940170940171	.955	.865
.958333333333333	.955	.838
.986111111111111	.909	.838
1.013513513513514	.864	.811
1.027799227799228	.864	.757
1.029437229437229	.864	.730
1.044563279857397	.864	.703
1.072268907563025	.818	.703
1.094581280788177	.818	.676
1.104355716878403	.818	.649

1.111455108359133	.773	.649
1.119429590017825	.773	.595
1.123106060606061	.727	.595
1.133928571428572	.682	.595
1.144957983193277	.591	.595
1.156862745098039	.545	.595
1.169540229885058	.545	.568
1.174442190669371	.545	.541
1.179144385026738	.545	.514
1.184659090909091	.500	.514
1.190524193548387	.500	.486
1.196774193548387	.500	.459
1.204166666666667	.455	.459
1.217069892473118	.409	.459
1.234115347018573	.364	.432
1.254545454545454	.364	.405
1.269696969696970	.318	.405
1.283422459893048	.318	.351
1.298573975044564	.318	.324

1.312805474095796	.318	.270
1.327956989247312	.318	.243
1.337719298245614	.318	.216
1.348471986417657	.318	.189
1.360752688172043	.318	.162
1.379761904761905	.273	.162
1.396428571428571	.227	.135
1.403703703703704	.182	.135
1.441798941798942	.136	.135
1.478835978835979	.091	.135
1.482120051085568	.091	.108
1.483314794215795	.091	.081
1.509792626728110	.091	.054
1.553571428571429	.000	.054
1.633540372670808	.000	.027
2.695652173913043	.000	.000

The test result variable(s): Alb:glob has at least one tie between the positive actual state group and the negative actual state group.

a. The smallest cutoff value is the minimum observed test value minus 1, and the largest cutoff value is the maximum observed test value plus 1. All the other cutoff values are the averages of two consecutive ordered observed test values.
